# Nationwide Surveillance and Molecular Characterization of Critically Drug-Resistant Gram-Negative Bacteria: Results of the Research University Network Thailand Study

**DOI:** 10.1128/AAC.00675-21

**Published:** 2021-08-17

**Authors:** Thitiya Yungyuen, Tanittha Chatsuwan, Rongpong Plongla, Sakawrat Kanthawong, Umaporn Yordpratum, Supayang P. Voravuthikunchai, Sarunyou Chusri, Dennapa Saeloh, Worada Samosornsuk, Nuntra Suwantarat, Romanee Chaiwarith, Surat Wannalerdsakun, Porpon Rotjanapan, Prawat Chantharit, Orawan Tulyaprawat, Iyarit Thaipisuttikul, Pattarachai Kiratisin

**Affiliations:** a Department of Microbiology, Faculty of Medicine, Siriraj Hospital, Mahidol University, Bangkok, Thailand; b Department of Microbiology, Faculty of Medicine, Chulalongkorn University, Bangkok, Thailand; c Antimicrobial Resistance and Stewardship Research Unit, Faculty of Medicine, Chulalongkorn University, Bangkok, Thailand; d Department of Medicine, Faculty of Medicine, Chulalongkorn University and King Chulalongkorn Memorial Hospital, Thai Red Cross Society, Bangkok, Thailand; e Department of Microbiology, Faculty of Medicine, Khon Kaen University, Khon Kaen, Thailand; f Division of Biological Science, Faculty of Science, Prince of Songkla University, Songkhla, Thailand; g Department of Internal Medicine, Faculty of Medicine, Prince of Songkla University, Songkhla, Thailand; h Faculty of Medical Technology, Prince of Songkla University, Songkhla, Thailand; i Department of Medical Technology, Faculty of Allied Health Sciences, Thammasat University, Pathum Thani, Thailand; j Department of Medicine, Chulabhorn International College of Medicine, Thammasat University, Pathum Thani, Thailand; k Department of Internal Medicine, Faculty of Medicine, Chiang Mai University, Chiang Mai, Thailand; l Department of Medicine, Faculty of Medicine, Naresuan University, Phitsanulok, Thailand; m Department of Medicine, Faculty of Medicine Ramathibodi Hospital, Mahidol University, Bangkok, Thailand

**Keywords:** drug resistance, Gram-negative bacteria, surveillance, *bla* gene, Thailand

## Abstract

A large-scale surveillance is an important measure to monitor the regional spread of antimicrobial resistance. We prospectively studied the prevalence and molecular characteristics of clinically important Gram-negative bacilli, including Escherichia coli, Klebsiella pneumoniae, Acinetobacter baumannii complex (ABC), and Pseudomonas aeruginosa, from blood, respiratory tract, urine, and sterile sites at 47 hospitals across Thailand. Among 187,619 isolates, 93,810 isolates (50.0%) were critically drug resistant, of which 12,915 isolates (13.8%) were randomly selected for molecular characterization. *E. coli* was most commonly isolated from all specimens, except the respiratory tract, in which ABC was predominant. Prevalence of extended-spectrum cephalosporin resistance (ESCR) was higher in *E. coli* (42.5%) than K. pneumoniae (32.0%), but carbapenem-resistant (CR)-K. pneumoniae (17.2%) was 4.5-fold higher than CR-*E. coli* (3.8%). The majority of ESCR/CR-*E. coli* and K. pneumoniae isolates carried *bla*_CTX-M_ (64.6% to 82.1%). *bla*_NDM_ and *bla*_OXA-48-like_ were the most prevalent carbapenemase genes in CR-*E. coli*/CR-K. pneumoniae (74.9%/52.9% and 22.4%/54.1%, respectively). In addition, 12.9%/23.0% of CR-*E. coli*/CR-K. pneumoniae cocarried *bla*_NDM_ and *bla*_OXA-48-like._ Among ABC isolates, 41.9% were extensively drug resistant (XDR) and 35.7% were multidrug resistant (MDR), while P. aeruginosa showed XDR/MDR at 6.3%/16.5%. A. baumannii was the most common species among ABC isolates. The major carbapenemase gene in MDR-A. baumannii/XDR-A. baumannii was *bla*_OXA-23-like_ (85.8%/93.0%), which had much higher rates than other ABC species. *bla*_IMP_, *bla*_VIM_, *bla*_OXA-40-like_, and *bla*_OXA-58-like_ were also detected in ABC at lower rates. The most common carbapenemase gene in MDR/XDR-P. aeruginosa was *bla*_IMP_ (29.0%/30.6%), followed by *bla*_VIM_ (9.5%/25.3%). The findings reiterate an alarming situation of drug resistance that requires serious control measures.

## INTRODUCTION

Critically drug-resistant Gram-negative bacilli (CDRGNB) constitute a significant cause of high morbidity and mortality worldwide. The most common CDRGNB in clinical practice include Escherichia coli, Klebsiella pneumoniae, Acinetobacter baumannii complex (ABC), and Pseudomonas aeruginosa. Among *E. coli* and K. pneumoniae, serious concerns are specifically for extended-spectrum cephalosporin-resistant (ESCR) and carbapenem-resistant (CR) isolates. The most important species of ABC is A. baumannii. Some isolates of ABC and P. aeruginosa demonstrate high resistance to several antimicrobial classes, defined as multidrug-resistant (MDR) and extensively drug-resistant (XDR) bacteria (1). Infections due to these CDRGNB likely contribute to significant long-term stays and high mortality rates (2). The World Health Organization announced that these bacteria were critical priorities for research and new antimicrobial agent development (3). A hospital surveillance system is a proactive approach to taking action on antimicrobial resistance and nosocomial infection. At the international level, surveillance data have been employed to monitor the emergence of resistant bacteria and prevent global spread.

Due to antimicrobial resistance, the mortality rate has strikingly exceeded 54 per 100,000 population in Thailand (4). The availability of over-the-counter drugs and overprescribing in hospitals of many antibacterial agents have provoked the situation. The survey revealed that 29.7% of Thai people were self-medicated with antibacterial agents, and most of them had symptoms resembling viral infection (5). The significant correlation between carbapenem consumption and the increase of CR-*E. coli* and CR-K. pneumoniae has also been demonstrated (6). While CDRGNB infection is on the rise in most Southeast Asian countries, large-scale surveillance of clinically significant isolates with molecular analysis remains very limited (7). This study aimed to conduct a nationwide prospective survey of CDRGNB prevalence in Thailand and investigate the molecular characteristics of beta-lactamase genes. CDRGNB isolates were classified into critically drug-resistant (CDR) groups, including CDR-*E. coli* (ESCR-*E. coli* and CR-*E. coli*), CDR-K. pneumoniae (ESCR-K. pneumoniae and CR-K. pneumoniae), CDR-ABC (MDR-ABC and XDR-ABC), and CDR-P. aeruginosa (MDR-P. aeruginosa and XDR-P. aeruginosa). The project was a part of the Research University Network Thailand's collaborative effort in partnership with government, military, and private hospitals to address the threatening health issues.

## RESULTS

### Prevalence of CDRGNB from clinical specimens.

There were 47 hospitals across all Thailand regions that participated in this study, including university, government, military, and private hospitals of various sizes and types of services, as shown in Table 1. A total of 187,619 isolates of four clinically significant Gram-negative bacteria (*E. coli*, K. pneumoniae, ABC, and P. aeruginosa) from blood, respiratory tract, urine, and sterile site samples were reported during the study period. ABC isolates were collected as a complex according to biochemical-based identification commonly used in hospital laboratories with limited species differentiation. These Gram-negative bacteria were mainly isolated from the respiratory tract (48.7%), followed by urine (32.2%), blood (13.7%), and sterile sites (5.4%). *E. coli* was the most predominant organism, followed by K. pneumoniae, ABC, and P. aeruginosa (Table 2). *E. coli* was most commonly isolated from blood, urine, and sterile sites, while ABC was most commonly isolated from the respiratory tract. K. pneumoniae was the second most common pathogen from all specimen types. Isolates in eight CDR groups comprised half of the study isolates, indicating the overall prevalence of CDRGNB at 50% from clinical specimens (Table 3). CDRGNB had high rates in all specimens, especially for the respiratory tract, urine, and sterile site samples, which were shown in over 50% of isolates. ESCR-*E. coli* was the most prevalent CDRGNB in overall specimens and was found at the highest rates from urine and blood, respectively, while ESCR-K. pneumoniae was most detected from the respiratory tract samples. XDR-ABC had a higher ratio over MDR-ABC and were most predominant in respiratory tract samples, followed by sterile sites. P. aeruginosa had less prevalence of MDR and XDR isolates than ABC and was also commonly found in the respiratory tract samples.

**TABLE 1:** Hospitals participating in this study

Type of hospital	Level of care	Size (no. of beds)	No.
Study center and study hubs			
University	Tertiary care	>1,000	4
		>500–1,000	2
		≤500	1
Satellite hospitals			
Government	Tertiary care	>1,000	2
		>500–1,000	19
		≤500	7
	Secondary care	≤500	8
Private	Secondary care	≤500	3
Military	Tertiary care	>500–1,000	1
Total			47

**TABLE 2:** Prevalence of Gram-negative bacteria in this study

Organism	No. of isolates from clinical specimen (%)				
	All	Blood	Respiratory	Urine	Sterile site
*E. coli*	63,991 (34.1)	14,548 (56.7)	6,760 (7.4)	39,540 (65.4)	3,143 (31.3)
K. pneumoniae	48,013 (25.6)	6,053 (23.6)	27,618 (30.2)	11,786 (19.5)	2,556 (25.4)
ABC	42,616 (22.7)	3,201 (12.5)	33,741 (36.9)	3,490 (5.8)	2,184 (21.7)
*P. aeruginosa*	32,999 (17.6)	1,853 (7.2)	23,330 (25.5)	5,647 (9.3)	2,169 (21.6)
Total	187,619 (100)	25,655 (100)	91,449 (100)	60,463 (100)	10,052 (100)

**TABLE 3:** Prevalence of CDRGNB in various specimens

Organism	CDR group	No. of isolates from clinical specimen (%)				
		All (*N* = 187,619)	Blood (*N* = 25,655)	Respiratory (*N* = 91,449)	Urine (*N* = 60,463)	Sterile site (*N* = 10,052)
*E. coli*	ESCR-*E. coli*	27,169 (14.5)	5,246 (20.4)	3,295 (3.6)	17,167 (28.4)	1,461 (14.5)
	CR-*E. coli*	2,404 (1.3)	274 (1.1)	403 (0.4)	1,566 (2.6)	161 (1.6)
K. pneumoniae	ESCR-K. pneumoniae	15,378 (8.2)	1,364 (5.3)	9080 (9.9)	4,160 (6.9)	774 (7.7)
	CR-K. pneumoniae	8,220 (4.4)	886 (3.5)	3893 (4.3)	2,860 (4.7)	581 (5.8)
ABC	MDR-ABC	15,222 (8.1)	969 (3.8)	11,975 (13.1)	1,481 (2.5)	797 (7.9)
	XDR-ABC	17,792 (9.5)	1,156 (4.5)	14,491 (15.9)	1,265 (2.1)	880 (8.8)
*P. aeruginosa*	MDR-*P. aeruginosa*	5,509 (2.9)	232 (0.9)	3,766 (4.1)	1,223 (2.0)	288 (2.9)
	XDR-*P. aeruginosa*	2,030 (1.1)	69 (0.3)	929 (1.0)	942 (1.6)	90 (0.9)
Total		93,823 (50.0)	10,169 (39.6)	47,832 (52.3)	30,664 (50.7)	5,032 (50.1)

**FIG 1:**
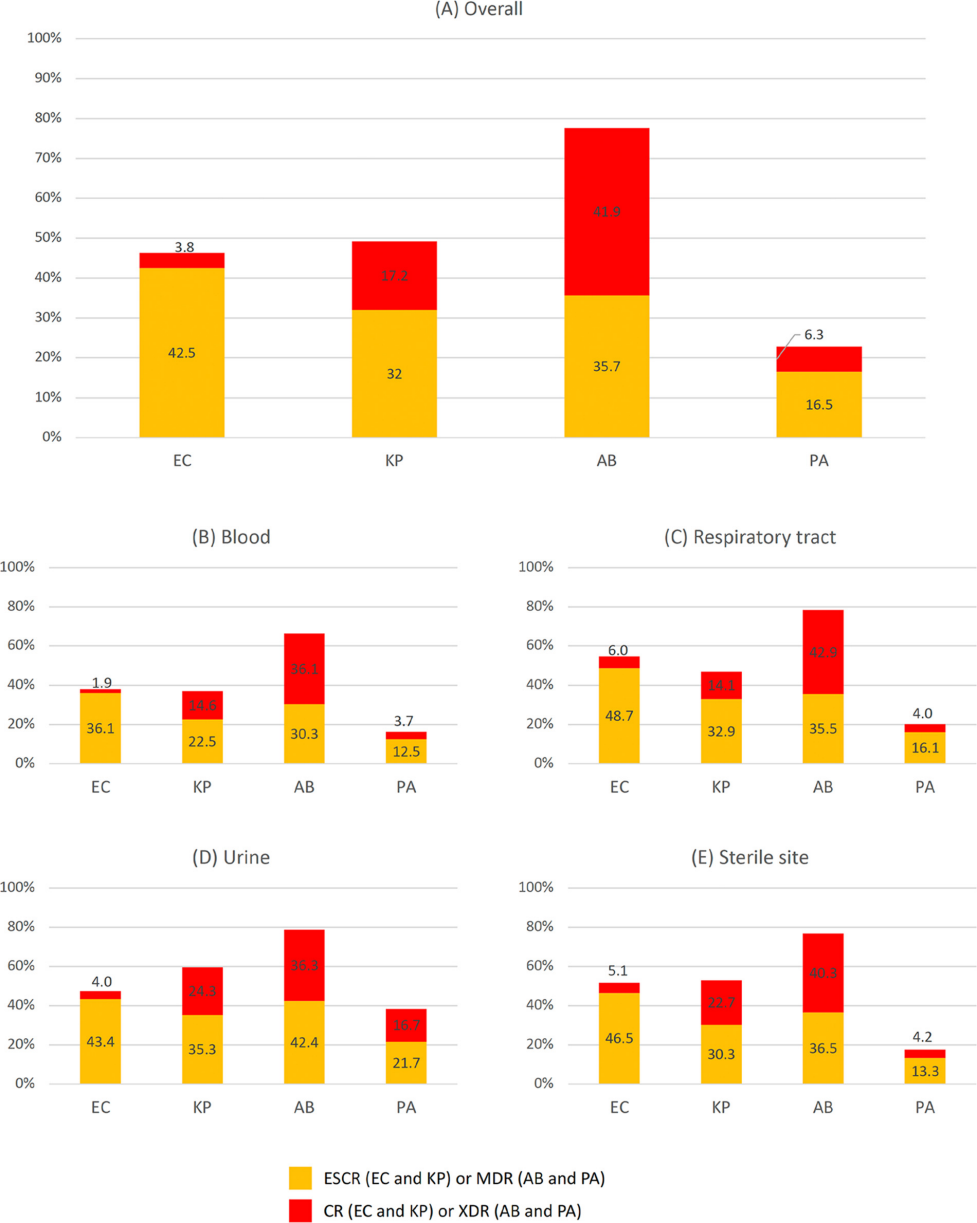
Prevalence of CDRGNB in different clinical specimens. Percentages of each CDRGNB group in overall (A), blood (B), respiratory tract (C), urine (D), and sterile site (E) samples are shown.

The distribution of each CDR group in clinical specimens is shown in Fig. 1. Overall, the prevalence of ESCR-K. pneumoniae was lower than ESCR-*E. coli*, but the CR-K. pneumoniae rate was 4.5-fold higher than that for CR-*E. coli*, resulting in a CDR-K. pneumoniae rate (49.2%) slightly higher than the CDR-*E. coli* rate (46.3%). A combination of ESCR-*E. coli* and CR-*E. coli* constituted more than half of *E. coli* isolates from the respiratory tract (54.7%) and sterile site (51.6%) samples and at high rates in urine (47.4%) and blood (38.0%) samples. ESCR-K. pneumoniae and CR-K. pneumoniae were detected in more than half of K. pneumoniae isolates from urine (59.6%) and sterile site (53.0%) samples and also at high rates in the respiratory tract (47.0%) and blood (37.1%) samples. CR-K. pneumoniae was at a higher rate than CR-*E. coli* in all specimen types. CDR-ABC were predominant among ABC isolates in all specimens, ranging from 66.4% to 78.7%. XDR-ABC had the highest rate in respiratory tract samples and higher rates than MDR-ABC in all specimen types except urine. MDR-P. aeruginosa and XDR-P. aeruginosa were less prevalent overall than other CDRGNB. CDR-P. aeruginosa was exceptionally high in urine samples, with a larger ratio of XDR-P. aeruginosa than in other specimens.

### Molecular characteristics of resistance genes among CDRGNB.

A total of 12,915 isolates, or approximately 13.8% of CDRGNB isolates, were randomly selected for resistance gene detection. The results of *bla* genes detected among these isolates are shown in Table 4. *bla*_CTX-M_ was highly prevalent among CDR-*E. coli* (65.9%) and CDR-K. pneumoniae (80.6%) at nearly similar rates between ESCR and CR groups of each organism. Among CR-*E. coli* isolates, *bla*_NDM_ was the predominant carbapenemase gene, followed by *bla*_OXA-48-like_. However, both *bla*_NDM_ and *bla*_OXA-48-like_ were common among CR-K. pneumoniae, with a slightly higher prevalence for *bla*_OXA-48-like_; 12.9% and 23.0% of CR-*E. coli* and CR-K. pneumoniae, respectively, carried both *bla*_NDM_ and *bla*_OXA-48_. Lesser numbers of CR-*E. coli* and CR-K. pneumoniae carried *bla*_VIM_, *bla*_IMP_, and *bla*_KPC_, which were slightly more common in CR-*E. coli* than in CR-K. pneumoniae. Based on the detection of *bla*_OXA-51-like_, 86.5% and 89.5% of ABC isolates were determined to be A. baumannii, and the rest were other species of ABC. *bla*_OXA-23-like_ was the most prevalent carbapenemase gene in both MDR-A. baumannii and XDR-A. baumannii isolates and was shown at a much higher rate than in other ABC species. Metallo-β-lactamase (MBL) genes (*bla*_IMP_ and *bla*_VIM_) and other OXA genes (*bla*_OXA-40-like_ and *bla*_OXA-58-like_) were also found with much less prevalence. Notably, *bla*_IMP_ and *bla*_OXA-58-like_ were more common among other ABC species than in A. baumannii. In addition to *bla*_OXA-51-like_, 67 (5.8%) and 38 (2.8%) isolates of MDR-A. baumannii and XDR-A. baumannii, respectively, carried both *bla*_OXA-23-like_ and *bla*_OXA-58-like_. Three MDR-A. baumannii isolates (0.3%), but not XDR-A. baumannii, had four *bla*_OXA_ genes (*bla*_OXA-23-like_, *bla*_OXA-40-like_, *bla*_OXA-51-like_, and *bla*_OXA-58-like_). However, all MDR-ABC and XDR-ABC isolates with an MBL gene carried either *bla*_IMP_ or *bla*_VIM_, i.e., none of them had both genes. *bla*_IMP_ was prominent at a similar rate in both MDR-P. aeruginosa and XDR-P. aeruginosa, followed by *bla*_VIM_, which was more pronounced in XDR-P. aeruginosa than in MDR-P. aeruginosa. Eight MDR-*P. aeruginosa* (0.9%) and 21 XDR-*P. aeruginosa* (3.9%) isolates had both *bla*_IMP_ and *bla*_VIM_.

**TABLE 4:** Molecular characteristics of *bla* genes among CDRGNB

CDR group (*n*)	% Detection								
	*bla*_CTX-M_	*bla*_KPC_	*bla*_NDM_	*bla*_IMP_	*bla*_VIM_	*bla*_OXA-23_	*bla*_OXA-40_	*bla*_OXA-48_	*bla*_OXA-58_
ESCR-*E. coli* (2,952)	64.6	—	—	—	—	—	—	—	—
CR-*E. coli* (722)	66.9	0.8	74.9	2.9	4.6	—	—	22.4	—
ESCR-K. pneumoniae (3,391)	79.9	—	—	—	—	—	—	—	—
CR-K. pneumoniae (1,591)	82.1	0.5	52.9	1.8	2.8	—	—	54.1	—
MDR-ABC									
A. baumannii (1,158)	—	—	—	2.0	2.9	85.8	1.0	—	7.9
Non-A. baumannii^*b*^ (181)	—	—	—	12.2	1.7	10.5	1.1	—	14.9
XDR-ABC									
A. baumannii (1,364)	—	—	—	0.8	1.3	93.0	0	—	3.9
Non-A. baumannii^*b*^ (160)	—	—	—	6.9	3.1	11.9	3.1	—	8.8
MDR-*P. aeruginosa* (854)	—	—	—	29.0	9.5	—	—	—	—
XDR-*P. aeruginosa* (542)	—	—	—	30.6	25.3	—	—	—	—

a —, not determined.

b Species of Acinetobacter baumannii complex other than A. baumannii based on the absence of *bla*_OXA-51_.

### Antimicrobial susceptibility of CDRGNB.

Table 5 shows susceptibility rates of CDRGNB groups to alternative antimicrobial agents. XDR-ABC and XDR-*P. aeruginosa* are not shown due to their resistance to all tested drugs. Carbapenems and amikacin were highly effective against ESCR-*E. coli* and ESCR-K. pneumoniae, and amikacin remained moderate against CR-*E. coli* and CR-K. pneumoniae. Piperacillin-tazobactam had good activity for ESCR-*E. coli* but not ESCR-K. pneumoniae. ESCR-*E. coli* and ESCR-K. pneumoniae isolates were moderately susceptible to netilmicin and cefoxitin, but most were resistant to ciprofloxacin, trimethoprim-sulfamethoxazole, and tetracycline. CR-*E. coli* and CR-K. pneumoniae were resistant to most drugs, except aminoglycosides may have moderate activity against them. A. baumannii isolates were slightly less susceptible than other ABC species, except for trimethoprim-sulfamethoxazole. All XDR-A. baumannii and 98.3% of MDR-A. baumannii isolates were resistant to at least a carbapenem, resulting in the rate of carbapenem resistance among A. baumannii of 77.0%. Among *P. aeruginosa*, 96.8% of MDR-*P. aeruginosa* and all XDR-*P. aeruginosa* isolates were resistant to at least a carbapenem, given *P. aeruginosa*'s carbapenem resistance rate of 22.3%. Amikacin showed moderate activity against MDR-ABC and MDR-*P. aeruginosa*. Trimethoprim-sulfamethoxazole and netilmicin had partial action against MDR-A. baumannii and MDR-*P. aeruginosa*, respectively.

**TABLE 5:** Antimicrobial susceptibility of CDRGNB

CDR group	% Susceptibility^*a*^												
	AMC	TZP	FOX	ETP	IMP	MEM	DOR	AMK	GEN	NET	CIP	SXT	TET
ESCR-*E. coli*	49.7	89.0	75.6	100	100	100	100	98.2	46.7	86.8	25.0	33.2	19.1
ESCR-K. pneumoniae	24.9	59.5	74.6	100	100	100	100	96.0	55.2	84.0	31.5	23.4	20.4
CR-*E. coli*	0.6	2.4	3.2	0.7	7.8	5.5	10.1	77.9	30.6	64.8	3.6	8.9	6.8
CR-K. pneumoniae	0.1	1.1	5.7	0.5	6.2	4.1	5.2	57.1	66.5	34.3	2.4	10.6	10.1
MDR-ABC													
A. baumannii	NA	2.5	NA	NA	3.3	3.0	3.0	55.3	26.5	NA	3.4	52.4	NA
Non-A. baumannii^*b*^	NA	12.7	NA	NA	8.2	9.2	5.5	57.5	26.5	NA	23.8	33.8	NA
MDR-*P. aeruginosa*	NA	25.0	NA	NA	4.4	5.9	6.8	65.3	19.9	43.3	12.7	NA	NA

a AMC (amoxicillin-clavulanate), TZP (piperacillin-tazobactam), FOX (cefoxitin), ETP (ertapenem), IMP (imipenem), MEM (meropenem), DOR (doripenem), AMK (amikacin), GEN (gentamicin), NET (netilmicin), CIP (ciprofloxacin), SXT (trimethoprim-sulfamethoxazole), TET (tetracycline), NA (not applicable).

b Species of Acinetobacter baumannii complex other than A. baumannii based on the absence of *bla*_OXA-51_.

## DISCUSSION

Infection due to CDRGNB has raised a serious concern worldwide and requires urgent solutions to reduce the wide spread of these organisms. Systematic surveillance of drug-resistant bacteria plays a vital role in monitoring and controlling the situation (8). This study conducted large-scale surveillance of four clinically significant Gram-negative bacteria in eight CDR groups. CDR-*E. coli* and CDR-K. pneumoniae were common causes of various infections, especially in the urinary tract, intra-abdominal area, and bloodstream, with high rates of extended-spectrum β-lactamase production and low susceptibility to fluoroquinolones, indicating multiple resistance mechanisms (9–11). The prevalence of CR-*E. coli* and CR-K. pneumoniae in Thailand has rapidly increased in the past decade. Studies in the late 2000s and early 2010s showed that *E. coli* and K. pneumoniae isolates from Thai patients were less than 1% and 5% resistant to carbapenems, respectively (12, 13). In this study, the CR-*E. coli* prevalence has risen to 3.8% and varied in different specimen types, ranging from 1.9% to 6.0%. An average proportion of CR-K. pneumoniae was 4.5-fold (ranging from 2.4- to 7.7-fold) higher than CR-*E. coli*. Therefore, within less than a decade, CR-*E. coli* and CR-K. pneumoniae prevalences in Thailand have risen more than three times. Inappropriate consumption of carbapenems was shown to contribute to an increased prevalence of CDRGNB, including CR-*E. coli* and CR-K. pneumoniae (6, 14). The increasing trends of CR-*E. coli* and CR-K. pneumoniae have also been reported from many countries in Southeast Asia, in which CR-K. pneumoniae often showed higher prevalence (15). Malaysia, Myanmar, Indonesia, and Vietnam were countries with a high prevalence of CR-*E. coli* (1.9% to 10.0%) and CR-K. pneumoniae (7.9% to 9.5%). Our study was among the most extensive and recent studies in Thailand. It revealed significantly higher CR-*E. coli* and CR-K. pneumoniae rates than what was shown in previous reports from Thailand and neighboring countries, raising a critical need for infection control actions. Aminoglycosides seemed to be the only class that is active for CR isolates but with modest activities. With a rise of CR isolates among *Enterobacteriaceae*, polymyxins, tigecycline, and fosfomycin were often choices for the alternative or last-line drug. However, these agents still have limitations due to toxicity or availability in different regions (16).

The most common carbapenemase genes in CR-*E. coli* and CR-K. pneumoniae detected in this study were *bla*_NDM_ and *bla*_OXA-48-like._ In contrast to North America, Latin America, some European countries, and China, where *bla*_KPC_ appears commonly, *bla*_KPC_ has rarely been detected in Thailand and Southeast Asia (17–19). *bla*_NDM_ has been endemic to South Asia and later widespread in Southeast Asia (15, 17). A study in Myanmar also revealed that all carbapenemase gene-carrying *Enterobacteriaceae* clinical isolates had *bla*_NDM_, of which *bla*_NDM-5_ was the most predominant (20). *bla*_NDM_ was first reported in Thailand in 2012 (21), and this study, conducted approximately 6 years later, showed a very high prevalence of *bla*_NDM_ in both CR-*E. coli* and CR-K. pneumoniae, suggesting a successful spreading of the gene in this country. Various mobile genetic elements mediate its rapid dissemination, such as transposons and conjugative plasmids. *bla*_NDM_ was also found in CR-*E. coli* and CR-K. pneumoniae isolates from the environment, likely related to contracting these organisms through traveling in the region of endemicity (18). While *bla*_IMP_ and *bla*_VIM_ were found at a low prevalence, *bla*_OXA-48-like_, a class D carbapenemase, was strikingly high among CR-*E. coli* and CR-K. pneumoniae. Originating in and primarily endemic to European and Middle East countries, *bla*_OXA-48-like_ and its derivatives have rapidly spread to Africa, South America, and Asia (17, 18). OXA-48 and OXA-48-like carbapenemases display unique characteristics of a modest hydrolytic activity for penicillins and carbapenems but not for cephalosporins, making it problematic for *in vitro* detection (22). Although OXA-48 usually confers weak resistance to carbapenems in *Enterobacteriaceae*, it may be cocarried with an MBL gene, e.g., *bla*_NDM_, as shown in this study, resulting in high-level carbapenem resistance (23).

CDR-ABC was shown to be the main portion of ABC isolates in all specimen types. The most common gene that mediates carbapenem resistance in ABC is *bla*_OXA_, which produces a class D OXA β-lactamase. The majority species of ABC was A. baumannii, based on the presence of intrinsic *bla*_OXA-51-like_ (24). Although *bla*_OXA-51-like_ may not be the most accurate marker for species confirmation, it is practical to screen for A. baumannii in an extensive survey. Besides chromosomally encoded *bla*_OXA-51-like_, we investigated three mainly plasmid-borne *bla*_OXA_ carbapenemase-related genes, *bla*_OXA-23-like_, *bla*_OXA-40-like_ (formerly known as *bla*_OXA-24_), and *bla*_OXA-58-like_, commonly present among ABC in Asian countries (25–28). All four OXA carbapenemases and their derivatives are transferable by a mobile genetic element. Either OXA-23 or OXA-51 is sufficient to confer carbapenem resistance in ABC, but generally, other carbapenemases and other mechanisms, e.g., efflux pump, often collectively coproduce high-level resistance. We showed that *bla*_OXA-23-like_ was most common among MDR-A. baumannii and XDR-A. baumannii but was less prevalent among other species of ABC. *bla*_OXA-40-like_ and *bla*_OXA-58-like_ are less common and usually confer low activity against carbapenems. This study reported that ABC isolates carry up to four *bla*_OXA_ genes, making them highly resistant to carbapenems. Although other resistance mechanisms were not investigated, it is likely that a combination of resistance determinants attributed to the extreme resistance phenotype in ABC.

The worldwide SENTRY surveillance program demonstrated that CDR-*P. aeruginosa* had a declining rate and was most susceptible to colistin, followed by amikacin (29). A previous study in Thailand among hospitalized patients during 2014 showed a higher rate of XDR-*P. aeruginosa* but a lower rate of MDR-*P. aeruginosa* compared to this study, suggesting a decreasing trend of XDR-*P. aeruginosa* but not MDR-*P. aeruginosa* (30). Amikacin was shown in the SENTRY report and this study to be most active against MDR-*P. aeruginosa*, yet with a modest susceptibility rate. Most CDR-*P. aeruginosa* isolates were resistant to carbapenem. The carbapenem resistance rate among *P. aeruginosa* isolates tends to be stable or decreasing over time in many regions (29, 31). *P. aeruginosa* often intrinsically harbors several β-lactam resistance mechanisms, including membrane modification, efflux pump, and AmpC enzyme hyperproduction (32). MBL enzymes were sporadically studied among carbapenem-resistant *P. aeruginosa*, of which IMP and VIM were the most common carbapenemases (33, 34). We reported that *bla*_IMP_ was detected at approximately similar rates in both MDR-*P. aeruginosa* and XDR-*P. aeruginosa*, while *bla*_VIM_ was detected 2.7-fold more often in XDR-*P. aeruginosa* than MDR-*P. aeruginosa*. In addition, XDR-*P. aeruginosa* isolates that carried both *bla*_IMP_ and *bla*_VIM_ genes were 4.3-fold more common than MDR-*P. aeruginosa*. Therefore, *bla*_VIM_ could contribute to extended resistance to multiple drugs among *P. aeruginosa* isolates, especially when cocarrying *bla*_IMP_. Despite a lesser proportion of resistant isolates than other CDRGNB, both MDR-*P. aeruginosa* and XDR-*P. aeruginosa* remain a severe threat because of their high resistance to commonly used antipseudomonal drugs and their critical role in nosocomial infection. Carbapenem resistance in *P. aeruginosa* is often mediated by MBL genes acquirable through a mobile plasmid and is significantly associated with high mortality (34).

This study has limitations in exploring specific and additional resistance genes and other mechanisms that could contribute to resistance due to a large number of study isolates. In addition, susceptibility to some alternative drugs, e.g., colistin, was not tested because the recommended testing methods were not available at all participating hospitals. Although A. baumannii is the most common species of ABC, this study used *bla*_OXA-51-like_ as a marker for A. baumannii but did not specifically differentiate all species. Therefore, phenotypic and genotypic resistance profiles of each species of ABC were not analyzed separately. These limitations would be further studied in selected groups to understand the evolving resistance among CDRGNB and their possible relatedness of resistance clones in various geographic regions. We emphasize in this report a high prevalence of CDRGNB in Thailand in eight CDR groups, raising concern for a severe threat in the Asia-Pacific region. These CDRGNB isolates are usually encountered in various infections, mainly associated with hospitalized patients. The threat of bacterial resistance should be critically addressed in Thailand and in the region for the necessity of appropriate antimicrobial prescription in clinical practice. In addition, the public policy regarding the self-consumption of antibacterial agents should be emphasized to reduce the risk of acquiring drug-resistant infections. Continuously prospective surveillance is an active measure to monitor and control the spread of resistance and to support the importance of antimicrobial stewardship programs. Further studies are recommended to investigate the impact of CDRGNB on various clinical aspects, such as community-acquired infections, nosocomial infections, and treatment outcomes. Multiple organizations and professionals are needed to build a scholarly society to prevent spreading resistance.

## MATERIALS AND METHODS

### Study design and study sites.

**FIG 2:**
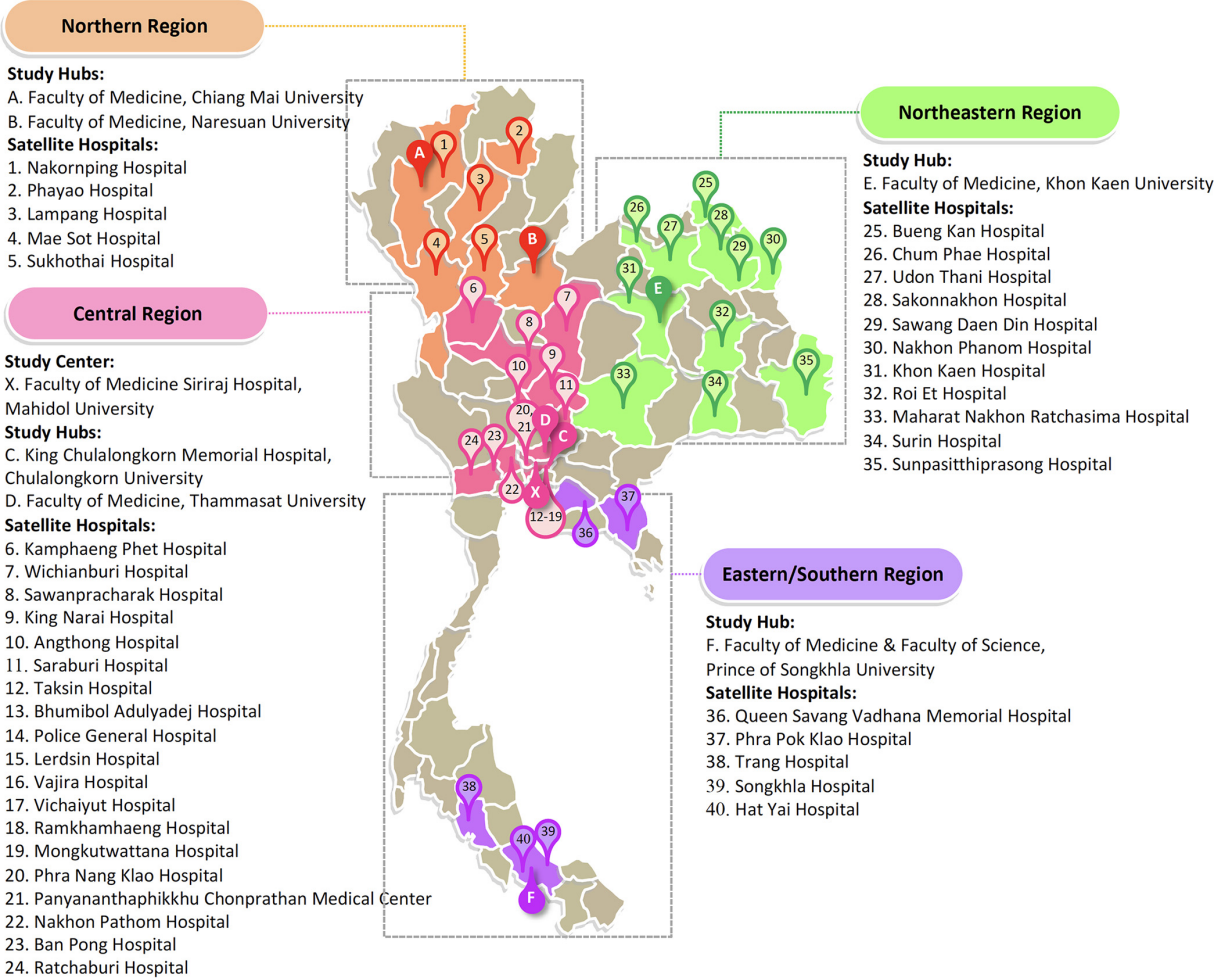
Location of study sites. Forty-seven hospitals across Thailand were enrolled in this study, including in the Northern (*n* = 7), Central (*n* = 22), Northeastern (*n* = 12), and Eastern/Southern (*n* = 6) regions. Hospitals were categorized into study center (*n* = 1), study hubs (*n* = 6), and satellite hospitals (*n* = 40).

Forty-seven hospitals participated as study sites were located across all regions of Thailand (Fig. 2). The Faculty of Medicine, Siriraj Hospital, Mahidol University (Bangkok), served as the study center, and an additional six major university hospitals served as the study hubs. The other 40 hospitals were designated satellite hospitals. Isolates and data from four clinically significant Gram-negative bacteria (*E. coli*, K. pneumoniae, ABC, and *P. aeruginosa*) were collected from blood, respiratory, urine, and sterile site (including cerebrospinal fluid, pleural fluid, abdominal fluid, joint fluid, and other fluids from sterile body parts) samples from patients between October 2017 and January 2019. Isolate collection was inclusive for all patient groups at each hospital without stratification according to disease onset or illness history. Isolates were evaluated for prevalence, antimicrobial susceptibility, and molecular characteristics. Each hospital performed bacterial identification and antimicrobial susceptibility testing, including quality control testing, at its laboratory based on standardized microbiological procedures, and additional external quality assessment was provided. Repeated isolates from the same patient were excluded. Random CDRGNB isolates were sent to a study hub or selected study centers for molecular study. The molecular study included the first three isolates of the month, if available, for each CDRGNB group in every clinical specimen from all hospitals for up to 12 months during the collection period. This procedure ensured that isolates were enrolled without selection bias of patient demography, geographic area, or time of collection. This study was approved by the Institution Review Board or Ethical Committee of all participating hospitals.

### Resistance definitions.

CDRGNB isolates were divided into eight CDR groups, including ESCR-*E. coli*, CR-*E. coli*, ESCR-K. pneumoniae, CR-K. pneumoniae, MDR-ABC, XDR-ABC, MDR-*P. aeruginosa*, and XDR-*P. aeruginosa*. As defined by the Centers for Disease Control and Prevention, ESCR isolates of *E. coli* and K. pneumoniae were resistant to at least one of these cephalosporin agents: ceftriaxone, cefotaxime, ceftazidime, and cefepime. CR isolates were resistant to at least one of these carbapenem agents: imipenem, meropenem, doripenem, and ertapenem (35). A. baumannii and *P. aeruginosa* were classified according to their resistance as MDR and XDR. MDR isolates were nonsusceptible to at least one agent in at least three antibacterial agent categories, and XDR isolates were nonsusceptible to at least one agent in all but two or fewer antimicrobial categories (1).

### Molecular characterization of *bla* genes.

Eight groups of CDRGNB from various specimens were collected for further molecular characterization. Each CDRGNB group was randomized monthly to ensure suitable distribution and that isolates collected each month during the study period were included. Bacterial DNA was extracted from colonies using the boiling method. PCR was used for the detection of beta-lactamase (*bla*) genes, including *bla*_CTX-M_, *bla*_KPC_, *bla*_NDM_, *bla*_IMP_, *bla*_VIM_, *bla*_OXA-23-like_, *bla*_OXA-40-like_, *bla*_OXA-48-like_, *bla*_OXA-51-like_, and *bla*_OXA-58-like_. The multiplex PCR primer sets (M1, M2, M3, and M4) and simplex PCR primer (S) were used to detect various specific genes, as detailed in Table 6. According to the corresponding reference, PCR was performed under the relevant conditions except where indicated, and the amplified targets were verified by DNA sequencing if necessary.

**TABLE 6:** PCR primers used in this study

Primer set	Target organism	Target gene	Primer sequence	Amplicon size (bp)	Reference
M1	ESCR-*E. coli* and ESCR-K. pneumoniae	*bla*_CTX-M_	(F) 5′-GCGATGTGCAGCACCAGTAA-3′	605	37
			(R) 5′-GGTTGAGGCTGGGTGAAGTA-3′		
		16S rRNA	(F) 5′-AGAGTTTGATCCTGGCTCAG-3′	802	38
			(R) 5′-GCGTGGACTACCAGGGTATC-3′		
M2	CR-*E. coli*, CR-K. pneumoniae, MDR-A. baumannii, XDR-A. baumannii, MDR-*P. aeruginosa* and XDR-*P. aeruginosa*	*bla*_IMP_16S rRNA	(F) 5′-GGAATAGAGTGGCTTAAYTCTC-3′(R) 5′-CCAAACYACTASGTTATCT-3′Similar to M1	188802	39 38
M3^*a*^	ESCR-*E. coli* and ESCR-K. pneumoniae	*bla*_NDM_	(F) 5′-GGTTTGGCGATCTGGTTTTC-3′	621	40
			(R) 5′-CGGAATGGCTCATCACGATC-3′		
		*bla*_KPC_	(F) 5′-CGTCTAGTTCTGCTGTCTTG-3′	798	40
			(R) 5′-CTTGTCATCCTTGTTAGGCG-3′		
		*bla*_OXA-48-like_	(F) 5′-GCGTGGTTAAGGATGAACAC-3′	438	40
			(R) 5′-CATCAAGTTCAACCCAACCG-3′		
M4	MDR-A. baumannii and XDR-A. baumannii	*bla*_OXA-23-like_	(F) 5′-GATCGGATTGGAGAACCAGA-3′	501	41
			(R) 5′-ATTTCTGACCGCATTTCCAT-3′		
		*bla*_OXA-__40__-like_	(F) 5′-GGTTAGTTGGCCCCCTTAAA-3′	246	41
			(R) 5′-AGTTGAGCGAAAAGGGGATT-3′		
		*bla*_OXA-51-like_	(F) 5′-TAATGCTTTGATCGGCCTTG-3′	353	41
			(R) 5′-TGGATTGCACTTCATCTTGG-3′		
		*bla*_OXA-58-like_	(F) 5′-AAGTATTGGGGCTTGTGCTG-3′	599	41
			(R) 5′-CCCCTCTGCGCTCTACATAC-3′		
S^*b*^	CR-*E. coli*, CR-K. pneumoniae, MDR-*P. aeruginosa*, XDR-*P. aeruginosa*, MDR-A. baumannii, and XDR-A. baumannii	*bla*_VIM_	(F) 5′-GATGGTGTTTGGTCGCATA-3′(R) 5′-CGAATGCGCAGCACCAG-3′	390	25

a The PCR conditions were 94°C for 5 min, 30 cycles of 95°C for 45 s/56°C for 45 s/72°C for 1 min, and 72°C for 5 min.

b Only for A. baumannii, the PCR conditions were 94°C for 5 min, 30 cycles of 94°C for 25 s/62°C for 40 s/72°C for 50 s, and 72°C for 6 min.

### Antimicrobial susceptibility testing.

Susceptibility testing was performed by using a standard disk diffusion assay or an automated system, either Sensititre (Thermo Fisher Scientific, OH, USA), Vitek2 (bioMérieux, Marcy-L'Etoile, France), or Phoenix (Becton, Dickinson, MD, USA), available at each study site. Results were interpreted according to the Clinical and Laboratory Standards Institute (CLSI) guidelines (36). All bacterial identification and antimicrobial susceptibility test results were submitted electronically and verified according to the resistance definitions.

## References

[1] Magiorakos AP, Srinivasan A, Carey RB, Carmeli Y, Falagas ME, Giske CG, Harbarth S, Hindler JF, Kahlmeter G, Olsson-Liljequist B, Paterson DL, Rice LB, Stelling J, Struelens MJ, Vatopoulos A, Weber JT, Monnet DL. 2012. Multidrug-resistant, extensively drug-resistant and pandrug-resistant bacteria: an international expert proposal for interim standard definitions for acquired resistance. Clin Microbiol Infect 18:268–281. 10.1111/j.1469-0691.2011.03570.x. 21793988

[2] Zhen X, Stålsby LC, Sun X, Hu X, Dong H. 2019. The clinical and economic impact of antibiotic resistance in China: a systematic review and meta-analysis. Antibiotics 8:115. 10.3390/antibiotics8030115. PMC6784351 31405146

[3] World Health Organization. 2017. Global priority list of antibiotic-resistant bacteria to guide research, discovery, and development of new antibiotics. WHO, Geneva, Switzerland. https://www.who.int/medicines/publications/WHO-PPL-Short_Summary_25Feb-ET_NM_WHO.pdf.

[4] Tangcharoensathien V, Sattayawutthipong W, Kanjanapimai S, Kanpravidth W, Brown R, Sommanustweechai A. 2017. Antimicrobial resistance: from global agenda to national strategic plan, Thailand. Bull World Health Organ 95:599–603. 10.2471/BLT.16.179648. 28804172 PMC5537745

[5] Chanvatik S, Kosiyaporn H, Lekagul A, Kaewkhankhaeng W, Vongmongkol V, Thunyahan A, Tangcharoensathien V. 2019. Knowledge and use of antibiotics in Thailand: a 2017 national household survey. PLoS One 14:e0220990. 10.1371/journal.pone.0220990. 31398242 PMC6688796

[6] Prakobsrikul N, Malathum K, Santanirand P, Chumnumwat S, Piebpien P, Montakantikul P. 2019. Correlation between antimicrobial consumption and the prevalence of carbapenem-resistant *Escherichia coli* and carbapenem-resistant *Klebsiella pneumoniae* at a university hospital in Thailand. J Clin Pharm Ther 44:292–299. 10.1111/jcpt.12791. 30578578

[7] Gandra S, Alvarez-Uria G, Turner P, Joshi J, Limmathurotsakul D, van Doorn HR. 2020. Antimicrobial resistance surveillance in low- and middle-income countries: progress and challenges in eight south Asian and southeast Asian countries. Clin Microbiol Rev 33:e00048-19. 10.1128/CMR.00048-19. 32522747 PMC7289787

[8] Perez F, Villegas MV. 2015. The role of surveillance systems in confronting the global crisis of antibiotic-resistant bacteria. Curr Opin Infect Dis 28:375–383. 10.1097/QCO.0000000000000182. 26098505 PMC4707665

[9] Jean SS, Coombs G, Ling T, Balaji V, Rodrigues C, Mikamo H, Kim MJ, Rajasekaram DG, Mendoza M, Tan TY, Kiratisin P, Ni Y, Weinman B, Xu Y, Hsueh PR. 2016. Epidemiology and antimicrobial susceptibility profiles of pathogens causing urinary tract infections in the Asia-Pacific region: results from the Study for Monitoring Antimicrobial Resistance Trends (SMART), 2010–2013. Int J Antimicrob Agents 47:328–334. 10.1016/j.ijantimicag.2016.01.008. 27005459

[10] Chang YT, Coombs G, Ling T, Balaji V, Rodrigues C, Mikamo H, Kim MJ, Rajasekaram DG, Mendoza M, Tan TY, Kiratisin P, Ni Y, Barry W, Xu Y, Chen YH, Hsueh PR. 2017. Epidemiology and trends in the antibiotic susceptibilities of Gram-negative bacilli isolated from patients with intra-abdominal infections in the Asia-Pacific region, 2010–2013. Int J Antimicrob Agents 49:734–739. 10.1016/j.ijantimicag.2017.01.030. 28435019

[11] Diekema DJ, Hsueh P-R, Mendes RE, Pfaller MA, Rolston KV, Sader HS, Jones RN. 2019. The microbiology of bloodstream infection: 20-year trends from the SENTRY antimicrobial surveillance program. Antimicrob Agents Chemother 63:e00355-19. 10.1128/AAC.00355-19. 31010862 PMC6591610

[12] Netikul T, Kiratisin P. 2015. Genetic characterization of carbapenem-resistant Enterobacteriaceae and the spread of carbapenem-resistant *Klebsiella pneumoniae* ST340 at a university hospital in Thailand. PLoS One 10:e0139116. 10.1371/journal.pone.0139116. 26407326 PMC4583293

[13] National Antimicrobial Resistance Surveillance Center, Thailand. 2019. Antibiograms. http://narst.dmsc.moph.go.th/antibiograms.html.

[14] Yang P, Chen Y, Jiang S, Shen P, Lu X, Xiao Y. 2018. Association between antibiotic consumption and the rate of carbapenem-resistant Gram-negative bacteria from China based on 153 tertiary hospitals data in 2014. Antimicrob Resist Infect Control 7:137. 10.1186/s13756-018-0430-1. 30479750 PMC6245771

[15] Malchione MD, Torres LM, Hartley DM, Koch M, Goodman JL. 2019. Carbapenem and colistin resistance in Enterobacteriaceae in Southeast Asia: review and mapping of emerging and overlapping challenges. Int J Antimicrob Agents 54:381–399. 10.1016/j.ijantimicag.2019.07.019. 31369812

[16] Morrill HJ, Pogue JM, Kaye KS, LaPlante KL. 2015. Treatment options for carbapenem-resistant Enterobacteriaceae infections. Open Forum Infect Dis 2:ofv050. 10.1093/ofid/ofv050. 26125030 PMC4462593

[17] Lee CR, Lee JH, Park KS, Kim YB, Jeong BC, Lee SH. 2016. Global dissemination of carbapenemase-producing *Klebsiella pneumoniae*: epidemiology, genetic context, treatment options, and detection methods. Front Microbiol 7:895. 10.3389/fmicb.2016.00895. 27379038 PMC4904035

[18] van Duin D, Doi Y. 2017. The global epidemiology of carbapenemase-producing Enterobacteriaceae. Virulence 8:460–469. 10.1080/21505594.2016.1222343. 27593176 PMC5477705

[19] Li Y, Sun QL, Shen Y, Zhang Y, Yang JW, Shu LB, Zhou HW, Wang Y, Wang B, Zhang R, Wang S, Shen Z. 2018. Rapid increase in prevalence of carbapenem-resistant Enterobacteriaceae (CRE) and emergence of colistin resistance gene *mcr-1* in CRE in a hospital in Henan. J Clin Microbiol 56:e01932-17. 10.1128/JCM.01932-17. 29386265 PMC5869811

[20] Sugawara Y, Akeda Y, Hagiya H, Sakamoto N, Takeuchi D, Shanmugakani RK, Motooka D, Nishi I, Zin KN, Aye MM, Myint T, Tomono K, Hamada S. 2019. Spreading patterns of NDM-producing Enterobacteriaceae in clinical and environmental settings in Yangon, Myanmar. Antimicrob Agents Chemother 63:e01924-18. 10.1128/AAC.01924-18. 30530602 PMC6395922

[21] Rimrang B, Chanawong A, Lulitanond A, Wilailuckana C, Charoensri N, Sribenjalux P, Phumsrikaew W, Wonglakorn L, Kerdsin A, Chetchotisakd P. 2012. Emergence of NDM-1- and IMP-14a-producing Enterobacteriaceae in Thailand. J Antimicrob Chemother 67:2626–2630. 10.1093/jac/dks267. 22796889

[22] Poirel L, Potron A, Nordmann P. 2012. OXA-48-like carbapenemases: the phantom menace. J Antimicrob Chemother 67:1597–1606. 10.1093/jac/dks121. 22499996

[23] Mohamed N, Said H, Hussin H, Abdul Rahman N, Hashim R. 2018. Carbapenem-resistant Enterobactericeae: clinico-epidemiological perspective. Trop Biomed 35:300–307. 33601804

[24] Turton JF, Woodford N, Glover J, Yarde S, Kaufmann ME, Pitt TL. 2006. Identification of *Acinetobacter baumannii* by detection of the *bla*_OXA-51-like_ carbapenemase gene intrinsic to this species. J Clin Microbiol 44:2974–2976. 10.1128/JCM.01021-06. 16891520 PMC1594603

[25] Wang TH, Leu YS, Wang NY, Liu CP, Yan TR. 2018. Prevalence of different carbapenemase genes among carbapenem-resistant *Acinetobacter baumannii* blood isolates in Taiwan. Antimicrob Resist Infect Control 7:123. 10.1186/s13756-018-0410-5. 30338061 PMC6182870

[26] Evans BA, Amyes SG. 2014. OXA β-lactamases. Clin Microbiol Rev 27:241–263. 10.1128/CMR.00117-13. 24696435 PMC3993105

[27] Bou G, Oliver A, Martinez-Beltran J. 2000. OXA-24, a novel class D β-lactamase with carbapenemase activity in an *Acinetobacter baumannii* clinical strain. Antimicrob Agents Chemother 44:1556–1561. 10.1128/AAC.44.6.1556-1561.2000. 10817708 PMC89912

[28] Heritier C, Poirel L, Lambert T, Nordmann P. 2005. Contribution of acquired carbapenem-hydrolyzing oxacillinases to carbapenem resistance in *Acinetobacter baumannii*. Antimicrob Agents Chemother 49:3198–3202. 10.1128/AAC.49.8.3198-3202.2005. 16048925 PMC1196226

[29] Shortridge D, Gales AC, Streit JM, Huband MD, Tsakris A, Jones RN. 2019. Geographic and temporal patterns of antimicrobial resistance in *Pseudomonas aeruginosa* over 20 years from the SENTRY antimicrobial surveillance program, 1997–2016. Open Forum Infect Dis 6:S63–S68. 10.1093/ofid/ofy343. 30895216 PMC6419917

[30] Palavutitotai N, Jitmuang A, Tongsai S, Kiratisin P, Angkasekwinai N. 2018. Epidemiology and risk factors of extensively drug-resistant *Pseudomonas aeruginosa* infections. PLoS One 13:e0193431. 10.1371/journal.pone.0193431. 29470531 PMC5823452

[31] European Centre for Disease Prevention and Control. 2019. Surveillance of antimicrobial resistance in Europe. Annual report of the European Antimicrobial Resistance Surveillance Network (EARSNet) 2019. https://www.ecdc.europa.eu/sites/default/files/documents/surveillance-antimicrobial-resistance-Europe-2019.pdf.

[32] Castanheira M, Mills JC, Farrell DJ, Jones RN. 2014. Mutation-driven β-lactam resistance mechanisms among contemporary ceftazidime-nonsusceptible *Pseudomonas aeruginosa* isolates from U.S. hospitals. Antimicrob Agents Chemother 58:6844–6850. 10.1128/AAC.03681-14. 25182652 PMC4249397

[33] Kazmierczak KM, Rabine S, Hackel M, McLaughlin RE, Biedenbach DJ, Bouchillon SK, Sahm DF, Bradford PA. 2016. Multiyear, multinational survey of the incidence and global distribution of metallo-β-lactamase-producing Enterobacteriaceae and *Pseudomonas aeruginosa*. Antimicrob Agents Chemother 60:1067–1078. 10.1128/AAC.02379-15. 26643349 PMC4750703

[34] Brink AJ. 2019. Epidemiology of carbapenem-resistant Gram-negative infections globally. Curr Opin Infect Dis 32:609–616. 10.1097/QCO.0000000000000608. 31567571

[35] Centers for Disease Control and Prevention. Patient safety atlas. https://www.cdc.gov/hai/surveillance/ar-patient-safety-atlas.html.

[36] Clinical and Laboratory Standards Institute. 2017. Performance standards for antimicrobial susceptibility testing, 27th ed. CLSI supplement M100. CLSI, Wayne, PA.

[37] Kiratisin P, Apisarnthanarak A, Laesripa C, Saifon P. 2008. Molecular characterization and epidemiology of extended-spectrum-beta-lactamase-producing *Escherichia coli* and *Klebsiella pneumoniae* isolates causing health care-associated infection in Thailand, where the CTX-M family is endemic. Antimicrob Agents Chemother 52:2818–2824. 10.1128/AAC.00171-08. 18505851 PMC2493136

[38] Srinivasan R, Karaoz U, Volegova M, MacKichan J, Kato-Maeda M, Miller S, Nadarajan R, Brodie EL, Lynch SV. 2015. Use of 16S rRNA gene for identification of a broad range of clinically relevant bacterial pathogens. PLoS One 10:e0117617. 10.1371/journal.pone.0117617. 25658760 PMC4319838

[39] Ellington MJ, Kistler J, Livermore DM, Woodford N. 2007. Multiplex PCR for rapid detection of genes encoding acquired metallo-beta-lactamases. J Antimicrob Chemother 59:321–322. 10.1093/jac/dkl481. 17185300

[40] Poirel L, Walsh TR, Cuvillier V, Nordmann P. 2011. Multiplex PCR for detection of acquired carbapenemase genes. Diagn Microbiol Infect Dis 70:119–123. 10.1016/j.diagmicrobio.2010.12.002. 21398074

[41] Woodford N, Ellington MJ, Coelho JM, Turton JF, Ward ME, Brown S, Amyes SG, Livermore DM. 2006. Multiplex PCR for genes encoding prevalent OXA carbapenemases in *Acinetobacter* spp. Int J Antimicrob Agents 27:351–353. 10.1016/j.ijantimicag.2006.01.004. 16564159

